# Does depressed persons with non-cardiovascular morbidity have a higher risk of CVD? A population-based cohort study in Sweden

**DOI:** 10.1186/s12872-019-1252-7

**Published:** 2019-11-21

**Authors:** Aysha Almas, Jette Moller, Romaina Iqbal, Andreas Lundin, Yvonne Forsell

**Affiliations:** 1 Department of Public Health Sciences, Karolinska Institutet, Widerströmska huset, 3rd floor, Tomtebodavägen 18 A, 171 77 Stockholm, Sweden; 2 Department of Medicine, Aga Khan University, Karachi, Pakistan; 3 Department of Community Health Sciences, Aga Khan University, Karachi, Pakistan

**Keywords:** Depression, Non-cardiovascular morbidity, Cardiovascular diseases, Morbidity

## Abstract

**Background:**

Depression often co-exists with non-cardiovascular morbid conditions. Whether this comorbidity increases the risk of cardiovascular disease has so far not been studied. Thus, the aim of this study was to determine if non-cardiovascular morbidity modifies the effect of depression on future risk of CVD.

**Methods:**

Data was derived from the PART study (acronym in Swedish for: Psykisk hälsa, Arbete och RelaTioner: Mental Health, Work and Relationships), a longitudinal cohort study on mental health, work and relations, including 10,443 adults (aged 20–64 years). Depression was assessed using the Major Depression Inventory (MDI) and self-reported data on non-cardiovascular morbidity was assessed in 1998–2000. Outcomes of CVD were assessed using the National Patient Register during 2001–2014.

**Results:**

Both depression (HR 1.5 (95% CI, 1.1, 2.0)) and non-cardiovascular morbidity (HR 2.0 (95% CI, 1.8, 2.6)) were associated with an increased future risk of CVD. The combined effect of depression and non-cardiovascular comorbidity on future CVD was HR 2.1 (95%, CI 1.3, 3.4) after adjusting for age, gender and socioeconomic position. Rather similar associations were seen after further adjustment for hypertension, diabetes and unhealthy lifestyle factors.

**Conclusion:**

Persons affected by depression in combination with non-cardiovascular morbidity had a higher risk of CVD compared to those without non-cardiovascular morbidity or depression alone.

## Background

Depressive disorders are a major cause of the non-fatal burden of diseases while ischemic heart disease and stroke are major causes of death [1]. Depression is strongly associated with other types of morbidity [2, 3]. Previous studies have shown this relation both specifically, as well as in combination, with other disorders [4, 5]. Further, depression is reported as a consequence, as well as, a risk factor for other morbidities [6]. For example, depression doubles the risk of future CVD [7–9] but also 40% of myocardial infarction patients report depression post-infarction [10]. As depression is linked both to morbidity and to CVD, the combined effect on future risk should be paid attention to in greater depth.

Depression increases the risk of CVD [7–9]. Established CVD-related morbidities (CVD risk factors) like hypertension and diabetes increase the risk of CVD [11]. In addition, other kinds of non-cardiovascular morbidity [12], such as rheumatoid arthritis [13] and osteoarthritis [14] also increase the risk of CVD [15]. Non-cardiovascular morbidity is a relatively new term, defined as comorbidities which are not established risk factors for CVD [16]. Generally, it includes respiratory, endocrine, nutritional, renal, hematopoietic, neurological as well as musculoskeletal conditions [17–19]. The number of comorbidities, and more importantly non-cardiovascular morbidities, have been reported to increase the severity of CVD (specifically heart failure) and this has been considered an important marker of prognosis in patients with heart failure [20, 21].

Depressive disorders are roughly two-times more prevalent among persons with diabetes, coronary artery disease, HIV infection, and stroke compared to persons not suffering from these diseases [6]. Further, approximately 12–15% of persons with multiple comorbid conditions also suffer from depression [22]. Coexistence of depression and other morbidities can lead to overexpression of somatic symptoms, like fatigue, thus complicating the diagnosis of depression [23]. Additionally, persons with depressive disorders exhibit reduced self-efficacy and “will to function” which might contribute to poor medication adherence and unhealthy lifestyle factors [24]. This results in reduced quality of life, higher costs, and worse health outcomes and requires extensive coordination across various sectors of the health services [3, 25].

The effect of the coexistence of depression and non-cardiovascular morbidity on the risk of CVD, has to our knowledge, been paid less attention. It can be hypothesized that the metabolic or immune-inflammatory pathways might be triggered in depressed patients by additional comorbid conditions which in turn affect the incidence of CVD [26]. This multifaceted coexistence of depression, other somatic and psychiatric morbidity and CVD is a challenge to the clinical care of depressed patients and the lack of clinical guidelines on management might lead to a worse prognosis and additional health problems [27, 28]. Considering this, we aim to increase the knowledge on this topic by determining if non-cardiovascular morbidity modifies the effect of depression on future risk of CVD. In this study we hypothesize that the risk of CVD is higher in depressed individuals with coexisting non-cardiovascular morbidity compared to those without.

## Methods

Data was derived from the PART study, a longitudinal cohort study conducted in Stockholm County, Sweden [29]. This study focused on mental health, work and relations among adults living in Stockholm. The data collection took place in 3 waves; 1998–2000-wave 1(W1), 2001–2003-wave 2(W2) and 2010-wave 3(W3). The data was linked to the Swedish health registers through the exclusive identifier “pin number”. All the participants gave written informed consent and answered a posted questionnaire. The questions focused on risks and protective factors for mental health as well as psychiatric rating scales. The Ethical Review Board at Karolinska Institutet, Stockholm, approved the study (case numbers 96–260, 01–218, 03–302, 2009/880–31, 2012/808–32).

The PART study initially intended to include 19,744 persons of which 19,457 could be reached, and 10,443 answered to the questionnaire at W1 (participation rate 53%). Internal missing was low due to repeated telephone calls. Non-response analyses from W1 was performed using available administrative registers-showing a slightly higher response in women, higher age groups, persons with higher income and education, being born in the Nordic countries and having no previous psychiatric illness [30]. In W2 the participation rate was 83% (*n* = 8622) and similar associations were seen [31]. A description of the study population according to depression status is shown in Table 1, data of 10,341 participants were available for analysis at baseline. For the purpose of this study participants with a current or previous history of myocardial infarction, angina or stroke (*n* = 267, 2.6%) were excluded leaving a total sample of 10,074 participants.

**Table 1:** Baseline characteristics of the participants overall, and stratified by depression status, *n* = 10,341

	Overall	Depressed	Non-depressed	*P* value^a^
	*N* = 10,341	*n* = 1488	*n* = 8832	
	n (%)	n (%)	n (%)	
Age group (in years)				
*20–30*	2595 (25.1)	432 (29.0)	2160 (24.5)	< 0.001
*31–40*	2476 (23.9)	364 (24.5)	2102 (23.8)	
*41–52*	2733 (26.4)	421 (28.3)	2308 (26.1)	
*> 52*	2534 (24.5)	271 (18.2)	2259 (25.6)	
Male gender	4620 (44.0)	463 (31.1)	4146 (46.9)	< 0.001
Socio-economic position^b^				
High and intermediate level salary	4702 (45.5)	493 (37)	4206 (50.2)	
Assistant –non manual workers	1438 (13.9)	222 (16.7)	1215 (14.5)	
Skilled workers	665 (6.4)	94 (7.1)	570 (6.8)	
Unskilled and semiskilled workers	1204 (11.6)	233 (17.5)	968 (11.6)	
Self-employed (other than professional)	729 (7.0)	80 (6.0)	649 (7.7)	
Students	435 (4.2)	122 (9.2)	311 (3.7)	
Retired	552 (5.3)	88 (6.6)	459 (5.5)	< 0.001
IHD	193 (1.9)	47 (3.2)	143 (1.6)	< 0.001
Stroke	86 (0.8)	23 (1.5)	63 (0.7)	0.003
Hypertension	716 (6.9)	126 (8.5)	590 (6.7)	0.01
Diabetes mellitus	221 (2.1)	38 (2.6)	181 (2.0)	0.20
Smoking ^c^				
Regular	1289 (12.4)	306 (24.5)	983 (13.6)	< 0.001
Occasional smoker	889 (8.6)	148 (11.9)	741 (10.3)	
Ex-smoker	2502 (24.1)	331 (26.5)	2171 (30.0)	
Never smoker	3796 (36.6)	462 (37.0)	3333 (46.1)	
Physical inactivity ^c^	3936 (46.3)	697 (55.9)	3237 (44.6)	< 0.001
Mean BMI (SD) in kg/m2	24.97 (3.9)	25.13 (4.4)	24.93 (3.7)	0.1
Hazardous alcohol use	2605 (25.2)	544 (36.6)	2060 (23.3)	< 0.001
Treated for psychiatric disorders	2365 (22.8)	821 (55.2)	1542 (17.5)	< 0.001
Non-cardiovascular morbidity	1356 (13.1)	323 (21.7)	1026 (11.6)	< 0.001
Cardiovascular diseases during follow-up	676 (6.5)	109 (7.3)	564 (6.4)	0.09
*Ischemic/hypertensive heart*	435 (4.2)	71 (4.8)	361 (4.1)	0.12
*Stroke*	298 (2.9)	53 (3.6)	245 (2.8)	0.05

^a^*P*-value using chi square test for comparison of prevalence between depressed and non-depressed

^b^Missing in SEP; 616 did not respond to the SEP question and did not report any routine activity

^c^Missing in smoking and physical inactivity = 1831; Out of these 1831, 1719 (16%) did not follow in W2, 146 did not respond to questions related to smoking and 112 did not respond to questions related to physical inactivity

### Depression

Depression was measured as the index disease and assessed using the Major Depression Inventory (MDI) from W1 and W2. Participants with MDI score above 20 in either of the two waves were considered as depressed. The MDI has shown high validity in both clinical and non-clinical samples, including the PART study [32–34].

### Non-cardiovascular morbidity

Non-cardiovascular conditions [35, 36] were derived from self-reports asking the following question; *Indicate if you are presently or previously been diagnosed or treated by a doctor or hospitalized for any of the following conditions?* neurological disorders (brain disease, spinal cord, nerve fibers, headache), endocrinological disorders (goiter, thyroid disease or metabolic disease), respiratory disorders (asthma, chronic bronchitis or other lung disease), gastrointestinal disorders (dyspepsia, bowel or liver disease), kidney or urinary tract disorders, genital disorders, serious infections, tumors (benign or malignant), rheumatological disorders, other heart diseases (except ischemic heart diseases), severe skin disorders and psychiatric disorders (other than depression). Presence of any of these was considered as non-cardiovascular morbidity.

### Cardiovascular disease

Cardiovascular disease (CVD) was evaluated using hospital discharge diagnoses classified according to the International Classification of Disease (ICD) in the National Patient Register (NPR) during 2001 to 2014, including the following diagnoses [37, 38]: ischemic/hypertensive heart disease; hypertensive diseases (ICD10: I11–13), ischemic heart diseases (ICD10: I20–25), heart failure (ICD10: I50), other peripheral vascular diseases, embolism and thrombosis (ICD10: I73–74) and stroke (ICD10: I60–67 and I69).

### Covariates

Age, gender, socioeconomic position (SEP) [39], hypertension, diabetes, smoking, hazardous alcohol use, body mass index (BMI) and physical inactivity were considered as covariates [40–42]. Physical inactivity and smoking were only recorded in W2 and hence have slightly higher levels of missing values. Age was divided into four categories: 20–30, 31–40, and 41–52 and > 52 years. SEP was measured by using the Nordic Standard Occupational Classification (NSOC) of 1989 and classified into five groups according to Statistics Sweden’s Goldthorpe-Eriksson classification scheme: high/intermediate level salaried employees; assistant non-manual employees; skilled workers; unskilled workers; and self-employed (including farmers). As a substantial number of participants (*n* = 2488) were not currently employed at the time of assessment, we used their daily reported activity and created additional categories; including those who were retired or students. Participants were considered as physically inactive if they reported exercising habitually less than three times a week. Self-reported measures on hypertension and diabetes mellitus were used from wave 1. They were questioned as follows; “Are you currently or have you previously been diagnosed or treated by a doctor or hospitalized for hypertension or diabetes.” Smoking habits were classified as regular smoker, occasional smoker, previous smoker and never smoker. Hazardous alcohol use was assessed in W1 or W2 by the Alcohol Use Disorders Identification Test (AUDIT) [43]. The AUDIT used a cutoff of ≥8 for men and ≥ 6 for women to classify alcohol intake into hazardous alcohol intake and no hazardous alcohol intake [44]. .Participants were considered to have hazardous alcohol intake for the wave they scored higher on the AUDIT.

### Statistical analyses

Mean (SD) was used to report continuous variables and frequency and percentage for categorical variables. Chi-square test was used to assess group differences.

Imputation of missing values for MDI was done using the mean value of the questions in the answered items. This was done when there were missing responses for one or two out of the 10 questions. Among those who participated in W1 or W2, if there were missing for more than two questions, the response was left as missing (0.2% (*n* = 23) in W1 and 1.4% (*n* = 121) in W2). A trivial percentage 0.2% (*n* = 21) of the participants did not respond to the questions on comorbid conditions. Smoking and physical inactivity were measured in W2 and as 16% (*n* = 1719) did not participate in W2, evidence was missing for them, additionally 146 participants did not respond to questions on smoking and 112 did not respond to questions on physical inactivity. No imputations were performed for variables other than MDI.

Cox regression models were built to estimate hazard ratios with corresponding 95% confidence intervals. Participants were followed from 1st of January 2001 to 31st of December 2014 and endpoints considered were time of IHD, stroke or death, or end of follow up. Time to event was calculated in years = time of event (date of admission to hospital for CVD event) -start of follow-up for those who had an event, and end of follow-up - start of follow-up for those who did not have a cardiovascular event. Cox regression models were adjusted for age, gender and SEP in model 1 and additionally for hypertension, diabetes mellitus, smoking, physical inactivity, BMI and hazardous alcohol intake in model 2.

Interaction was measured on an additive scale as it is appropriate for assessing public health importance [45]. Four dummy variables of depression and non-cardiovascular morbidity were created to measure interaction. These were; no depression and no non-cardiovascular morbidity (reference category), depression with no non-cardiovascular morbidity, no depression but with non-cardiovascular morbidity, and depression with non-cardiovascular morbidity. Synergy index (S) is used to measure the effect of binary interaction among risk factors on the outcome [46] and to determine if additive interaction was present. S = 1 means no interaction or exactly additivity; S > 1 means positive interaction or more than additivity; S < 1 means negative interaction or less than additivity. S can range from 0 to infinity [47]. SAS 9.3 and SPSS version 19.11 and were used for the statistical analyses.

## Results

The overall prevalence of depression was 14.6% and of non-cardiovascular morbidity 13.1%. Among those who were depressed, 21.7% had non-cardiovascular morbidity. At follow up 6.5% had a CVD diagnosis; 4.2% had ischemic heart diseases and 2.9% had stroke.

Distribution of the specific non-cardiovascular conditions, stratified by depression status, is shown in Table 2. The most common non-cardiovascular conditions reported were respiratory disorders: 3.1%; followed by endocrinological disorders (other than diabetes mellitus): 1.8%; and rheumatologic disorders: 1.5%. A small proportion of the participants (1.6% (*n* = 164)) also reported psychiatric disorders (other than depression).

**Table 2:** Distribution of specific non-cardiovascular morbidity by depression status, *n* = 10,074

	All	Depressed	Not depressed	*P* value
	*N* = 10,074	*n* = 1488	*n* = 8832	
	n (%)	n (%)	n (%)	
Neurological disorders	137 (1.4)	59 (43.0)	78 (56.9)	< 0.001
Endocrinological disorders (other than DM)	186 (1.8)	41 (22.0)	145 (78.0)	0.001
Respiratory disorders	312 (3.1)	82 (26.3)	230 (73.7)	< 0.001
Gastrointestinal disorders				
Dyspepsia	105 (1.1)	45 (42.9)	60 (57.1)	< 0.001
Bowel disorders	89 (0.9)	27 (30.0)	62 (69.7)	< 0.001
Liver disorders	22 (0.2)	8 (36.4)	14 (63.6)	0.002
Nephrological (kidney) disorders	97 (1.0)	25 (25.8)	72 (74.2)	0.001
Genito urinary disorders	115 (1.2)	30 (26.1)	85 (73.9)	< 0.001
Tumors				
Benign	36 (0.4)	09 (25.6)	27 (75.0)	0.04
Malignant	39 (0.4)	10 (25.6)	29 (74.4)	0.02
Rheumatological disorder	138 (1.5)	35 (25.4)	103 (74.6)	< 0.001
Dermatological disorder	69 (0.7)	14 (20.3)	55 (79.7)	0.02
Other heart disorders (except ischemic heart diseases)	43 (0.5)	11 (25.6)	32 (74.4)	0.03
Severe infection	46 (0.5)	18 (39.2)	28 (60.8)	< 0.001

Depression, as reported previously [7], and non-cardiovascular morbidity were associated with increased risk of CVD (HR 1.5 (95% CI 1.2, 2.0) and HR 2.0 (95% CI 1.8, 2.6) respectively).

The interaction effect of non-cardiovascular morbidity and depression on risk of CVD is demonstrated in Table 3, showing hazard ratios for the combined effect of depression and non-cardiovascular morbidity on the risk of future CVD. Among those who were depressed without and with non-cardiovascular morbidity, the HR (95% CI) for future CVD were 1.4 (95% CI 1.1, 2.0) and 2.1 (95% CI 1.3, 3.4) respectively, adjusted for age, gender and SEP. The associations remained statistically significant after further adjustment for diabetes, hypertension, physical inactivity, smoking, BMI and hazardous alcohol use. The synergy index for additive interaction was 1.5 (95% CI 1.0, 2.2) after considering all covariates.

**Table 3:** Effect of depression and non-cardiovascular morbidity on the risk of CVD, *n* = 10,074

	Model 1^a^ *n* = 9458 HR (95% CI)		Model 2^b^ *n* = 7627 HR (95% CI)	
	Non-cardiovascular morbidity		Non-cardiovascular morbidity	
Depression	No	Yes	No	Yes
No	1 (Ref)	1.5 (1.1,2.0)	1 (Ref)	1.4 (1.0,2.0)
Yes	1.4 (1.0,2.0)	2.1 (1.4,3.4)	1.3 (1.0,2.0)	2.0 (1.1,3.3)
Synergy index (95% CI)	1.2 (0.8,1.7)		1.5 (1.0,2.2)	

^a^ Model 1 adjusted for age, gender and socioeconomic position,

^b^ Model 2 adjusted for age, gender, socioeconomic position, diabetes, hypertension, physical inactivity, BMI, smoking and hazardous alcohol consumption

## Discussion

In this large population-based study we found that coexistence of depression and non-cardiovascular morbid conditions doubled the risk of CVD. This additive interaction between depression and non-cardiovascular morbid conditions is also supported by the synergy index. The effect remained after adjusting for unhealthy lifestyle factors and other previously reported cardiovascular risk factors.

Several studies demonstrate an association between depression and CVD [7–9] as well as with other morbid conditions [3, 6, 48]. Studies have additionally shown that having an increased level of non-cardiovascular morbidity worsens the outcome of cardiovascular diseases like myocardial infarction and heart failure [49–52]. .However, the results of these studies are limited by the cross-sectional designs and have not assessed the interplay with depression. There are some studies which have focused on depression with specific comorbidities leading to CVD. For example, in a longitudinal study, patients with depression and rheumatoid arthritis were 1.4 times more likely than non-depressed rheumatoid arthritis patients to have myocardial infraction at follow-up [53]. To the best of our knowledge, only one previous study has focused on depression, comorbidity and CVD. The study included 6394 subjects who participated in the First National Health and Nutrition Examination Survey (NHANES I) conducted between 1971 and 1975 and later followed-up between 1982 and 1984 [54]. Depression and prevalent chronic medical conditions were assessed at baseline and participants were followed up for CVD mortality and incident medical conditions. The results showed that having more severe depressive symptoms increased the risk of CVD mortality at follow-up (HR 1.5 (95% CI 1.2, 1.8)), however the risk attenuated when taking into account demographics, lifestyle behaviors, prevalent and incident chronic medical conditions (HR 1.1 (95% CI 0.9, 1.4)). The study therefore concluded that the association between depression and CVD mortality was partially mediated by prevalent/incident comorbidity. Our study is in partial agreement with this study, showing that depression with coexisting comorbidity increased the risk of CVD. The reason for only partial agreement can be that our study mainly assessed non-cardiovascular morbidity at baseline and found that its existence in the depressed increased the incidence of CVD (HR 2.1 (95% CI 1.3–3.4)). Additionally, there are some important differences that make it difficult to compare results, including differences in assessment of depression, type of comorbid conditions and CVD outcome. Also, we considered non-cardiovascular morbidity as an effect modifier whereas the former study adjusted for it in the analyses. This led to underestimation of the risks in the former study as adjusting for comorbid conditions might have accounted for most of the association between depression and CVD. Also, the former study included conventional CVD risk factors (e.g. diabetes, hypertension) in their assessment of comorbidity, whereas we considered only non-cardiovascular ones.

One potential explanation for our increased risk of CVD in depressed people with coexisting non-cardiovascular morbidity could be poorer treatment adherence [55]. Lack of coordination between physicians may contribute to patients’ non-compliance of drugs prescribed for both comorbid and depressive conditions [56]. Therefore, a combined, integrated clinical care and self-management program to prevent and manage multi-morbidity including depression is needed [57]. Also, due to polypharmacy for different comorbid conditions, there might be drug-drug interactions leading to adverse clinical outcomes [58]. The underlying mechanisms shared between depression and comorbidity leading to CVD are not well established. Inflammation indicated by circulating levels of interleukin-6, C-reactive protein, and fibrinogen may be an important biological mechanism through which chronic medical conditions are linked to disorders in later life [59]. Inflammation is also an underlying shared mechanism between depression and CVD [60]. It might be that in depressed people, an additional comorbid condition triggers a pathway of inflammation contributing to the risk of CVD [26].

The strengths of this study lie in its longitudinal design, population-based large sample and use of validated instruments for assessing depression and register-based CVD outcomes. Our study has also some limitations that should be acknowledged. The baseline response rate was low in wave 1 (53%) [30, 31]. People severely affected by psychiatric disorders including major depression probably did not participate and this might have led to an underestimation of the magnitude of the problem and resulted in limited external validity. Hence, results cannot be generalized to patients with severe psychiatric disorders. Although we did have a relatively large proportion (5 .6%, *n* = 587) of participants with severe depression in the PART cohort [7], we did not have enough power to analyze a potential dose-response relationship. One limitation is the assessment of comorbidity which was based on self-reports and hence prone to recall bias due to lack of memory and may have resulted in non-differential misclassification thus moving the estimates towards the null. Previous studies have shown high validity of self-reporting for assessing comorbidity [61] but more objective measurements of the comorbid conditions with objective diagnostic scales [62], or diagnostic tests [63], could have further strengthened the assessments and improved the validity of the study. Since both depression and non-cardiovascular morbidity were measured at the same point in time, we cannot clarify the temporality between the two exposures. Establishing temporality is a challenge as DSM-5 manual also debates temporality and has categorized depression with chronic conditions separately [64]. Although this study was able to adjust for many covariates, it did not offer the possibility to adjust for mediators like dyslipidemia, blood glucose, type and use of antidepressants and treatment compliance. Information on antidepressant use is important in this relation as antidepressants like tricyclic antidepressants are known to increase the risk of cardiac arrhythmia which in turn may contribute to the studied CVDs [65, 66]. However instinctive expectation of effect decomposition of mediators might nullify the total effect of the exposure and outcome [67]. Although we were able to show a doubled risk for CVD when both depression and non-cardiovascular morbidity were present, the synergy index > 1 indicated additive interaction but due to the borderline confidence interval, the results need to be replicated in future larger studies.

## Conclusion

In this study, we showed that persons affected by depression in combination with non-cardiovascular morbidity had a higher risk of CVD compared to those without non-cardiovascular morbidity or depression alone. The additive interaction we found needs to be replicated in future longitudinal studies and these should also be designed to address the temporality in the occurrence of depression and comorbid conditions. From a clinical perspective, future research needs to address the identification of concomitance and to design interventions encompassing simultaneous treatment of comorbidities and depression to prevent future CVD.

## Data Availability

Data are ethically restricted for patient privacy concerns. However, de identified, participant level data can be obtained pending ethical approval. Please send requests for a Minimal dataset to Dr. Yvonne Forsell; yvonne.forsell@aku.edu.

## Abbreviations

CVD: Cardiovascular disease
ICD: International classification of disease
MDI: Major depression inventory
NSOC: Nordic standard occupational classification
PART: Psykisk hälsa, Arbete och RelaTioner, acronym in Swedish for: mental health, work and relationships
SEP: Socioeconomic position
